# Expression level and clinical significance of NBAT-1 in human cancers: a systematic review and meta-analysis

**DOI:** 10.1186/s12885-023-11770-w

**Published:** 2024-01-19

**Authors:** Yang Yu, Kedi Fan, Tingting Ni, Xun Lei Zhang, Xiaoqin Su, Lei Yang

**Affiliations:** 1 Department of Oncology, Affiliated Tumour Hospital of Nantong University, Nantong, Jiangsu Province People’s Republic of China; 2 Department of Medical School, Nantong University, Nantong, Jiangsu Province People’s Republic of China

**Keywords:** Neoplasms, NBAT-1, Meta-analysis, Prognosis

## Abstract

**Purpose:**

There is an aberrant expression of NBAT-1 in various human cancers, which was proven to limit the proliferation, invasion, and metastasis of tumour cells via multiple approaches. Most existing research focuses on sample size and discrete outcomes. Thus, a quantitative meta-analysis was performed to elucidate the prognostic value of lncRNA NBAT-1 expression in cancer patients.

**Materials and methods:**

Using Web of Science and PubMed, two researchers independently identified relevant studies to explore the association between the pathological features of human cancers and NBAT-1 expression levels. Then two scholars conducted literature screening according to exclusion criteria and admission criteria, and finally conducted statistical analysis through data extraction with StataSE 12.0.

**Results:**

A total of 12 eligible studies with 1600 patients were included in the meta-analysis eventually. It is indicated that the low expression level of lncRNA NBAT-1 was closely related to distant metastasis [RR = 0.50, 95% CI (0.33, 0.76), and *P* = 0.00], deep tumour invasion [RR = 0.62, 95% CI (0.49,0.80), and *P* = 0.00], poor histological grade [RR = 0.68, 95% CI (0.57, 0.81), and *P* = 0.00], advanced TNM stage [RR = 0.66, 95% CI (0.55, 0.79), and *P* = 0.00], large tumour volume[RR = 0.72, 95% CI (0.55, 0.93), and *P* = 0.01], and lymph node metastasis [RR = 0.62, 95% CI (0.46, 0.84), and *P* = 0.00], suggesting that it may serve as biomarkers for patients with poor prognosis.

**Conclusion:**

Reduced expression of NBAT-1 can predict poor prognosis in several cancers, as found in the meta-analysis, demonstrating that NBAT-1 can serve as a promising prognostic factor of human cancers.

## Introduction

Cancer is one of the leading causes of death and one of public health barriers that threatens human health worldwide. According to estimates by the World Health Organization (WHO) in 2019, cancer is the first or second leading cause of death in people under 70 years of age in 112 out of 183 countries, while it ranks third or fourth in another 23 countries [1]. In the latest Global Cancer Statistics, it is estimated that there will be 19.3 million new cases and 10 million cancer deaths worldwide in 2020 [1]. Therefore, there is an urgent need to find a new effective biomarker that can be used to predict the prognosis of different types of cancer and improve the survival outcomes of cancer patients.

Over 75% of the genome is activated and transcribed into noncoding RNAs (ncRNAs) and only 2% is transcribed into proteins according to the high-throughput sequencing of the genome and transcriptome [2]. Although the noncoding transcriptome was first regarded as transcriptional noise, increasing research has demonstrated that lncRNAs are essential in the pathological and physiological processes at various levels [3–5]. Long noncoding RNAs (lncRNAs) refer to a new heterogeneous category of ncRNAs with a length of over 200 nucleotides. Through chromatin remodelling, histone methylation, genetic imprinting, as well as transcriptional and post-transcriptional regulation, lncRNAs can control gene expression [4, 6]. For instance, in the nucleus, lncRNAs bind to chromatin-modifying complexes, transcription factors, and other DNA-binding proteins, which can lead to epigenetic alterations [7–9]. In the cytoplasm, lncRNAs function as miRNA sponges to regulate the translation and degradation of mRNAs [10, 11]. In addition, lncRNAs participate in ubiquitination, phosphorylation, and other protein modifications [12]. A growing body of work has shown that the dysregulation of lncRNA in various cancers is related to various physiological and pathological processes such as tumor cell proliferation, migration, invasion, angiogenesis and tumor progression [13–16]. LncRNAs have also been evaluated by numerous studies as independent novel biomarkers for cancer diagnosis and prognosis prediction [17–19].

NBAT-1 (a lncRNA neuroblastoma-associated transcript-1), which maps to the 6p22 locus, was first identified by Gaurav Kumar Pandey as a biomarker to predict the clinical results of neuroblastoma [20]. There is an aberrant expression of NBAT-1 in various human cancers, which was proven to limit the proliferation, invasion, and metastasis of tumour cells via multiple approaches. We now summarize the potential targets and pathways of NBAT-1 as follows (Table 1).

**Table 1:** Summary of potential targets and pathways of NBAT-1

Cancer type	Expression	Potential target	Pathway	Biological Behaviour	References
Neuroblastoma	Downregulated	SOX9 and NRSF/REST	NBAT-1/NRSF/REST pathway; NBAT-1/EZH2/target genes(SOX9、OSMR、VCAN) pathway	Cell proliferation, invasion and neuronal differentiation	[20]
Neuroblastoma	Downregulated	MYCN	NBAT-1/CASC15/MYCN/USP36/COL18A1 axis	Cell proliferation	[21]
Neuroblastoma	Downregulated	CRM1, MDM2	p53/NBAT-1/CRM1, MDM2 axis	Chemotherapeutic response	[22]
Glioma	Downregulated	miR‐21	NBAT-1/miR‐21/SOX7 axis	Cell proliferation, migration and invasion	[23]
Glioma	Downregulated	Akt	NBAT-1/Akt pathway	Cell proliferation	[24]
Lung cancer	Downregulated	RAC1	NBAT-1/RAC1 pathway	Cell proliferation and apoptosis	[25]
Non-small cell lung cancer	Downregulated	ATG7	NBAT1/PSMD10/ATG7pathway	Autophagy	[26]
Osteosarcoma	Downregulated	miR-21	NBAT-1/miR-21/PTEN, PDCD4, TPM1 and RECK axis	Cell proliferation, migration and invasion	[27]
Gastric cancer	Downregulated	Sox9	NBAT-1/Sox9 feedback regulation	Cell proliferation, apoptosis, angiogenesis, migration and invasion	[28]
Gastric cancer	Downregulated	PTEN	NBAT-1/PTEN axis	Cell proliferation and apoptosis	[29]
Breast cancer	Downregulated	Dkk1	NBAT-1/EZH2/DKK1 pathway	Migration and invasion	[30]
Epithelial ovarian cancer	Downregulated	ERK1/2 and AKT	NBAT-1/ERK1/2 and AKT signalling pathways	Cell proliferation, migration and invasion	[31]
Hepatocellular carcinoma	Downregulated	c-Myc	NBAT-1/IGF2BP1 and c-Myc	Cell proliferation and apoptosis	[32]
Bladder cancer	Downregulated	EMT	NBAT-1/EMT pathway	Cell proliferation, migration and apoptosis	[33]
Colorectal Carcinoma	Downregulated	miR-4504	NBAT-1/miR-4504/WWC3/LATS1/YAP axis	Chemoresistance of CRC	[34]
Endometrial Cancer	Downregulated	miR-21-5p	NBAT-1/miR-21-5p/PTEN axis	Cell Metastasis, apoptosis and invasion	[35]
Renal carcinoma	Downregulated	miR-346	NBAT1/miR-346/GSK-3β axis	cell proliferation, migration, and invasion	[36]

For nervous system, NBAT1 affects the biological behaviour of tumours through different pathways. In neuroblastoma, NBAT-1 affects target genes relevant to cell invasion and proliferation in epigenetics by acting as a scaffold for EZH2, and by mechanically inhibiting the NRSF/REST pathway the neural differentiation is controlled by it as well [20, 37]. In addition, in neuroblastoma, NBAT-1/CASC15/MYCN/USP36/COL18A1 controls a new oncogenic pathway, as revealed by Prasanna Kumar Juvvuna [21]. Additionally, Sanhita Mitra proved that NBAT-1, which can act as a p53-responsive lncRNA, is resistant to genotoxic drugs by altering CRM1 and MDM2 function, thus accelerating the accumulation of p53 in cytoplasm and the loss from mitochondrial and nuclear compartments [22]. In glioma, the results of a study by Ning Guan suggested that NBAT-1 may up-regulate SOX7 by inhibiting miR-21 to suppress the invasion, migration, and proliferation of glioma cells [23]. Moreover, J. LIU demonstrated that NBAT-1 can affect the prognosis and the malignant degree of gliomas by regulating Akt [24].

NBAT1 plays a similar role in the respiratory system. In lung cancer, as revealed by T. Lei, NBAT-1 is downregulated and can affect the cell cycle and the apoptosis and proliferation of cells by downregulating the expression level of RAC1 [25]. Furthermore, Tianliang Zheng proved that NBAT-1 could inhibit autophagy by interacting with PSMD10 and promoting its degradation. Therefore, in non-small cell lung cancer, it can inhibit the activeness of HSF1 and PSMD10 in the ATG7 promoter, thus inhibiting ATG7 transcription [26].

It has also been reported that NBAT-1 affected gastric cancer, hepatocellular carcinoma and colorectal carcinoma in the digestive system. By enhancing the proteasome-dependent degradation and polyubiquitination of Sox9, NBAT-1 interacts with it to reduce the stability. Also, Sox9 can use the NBAT-1 promoter to suppress its transcription. The suppressive effects of the negative feedback loop of Sox9 and NBAT-1 kept being enhanced [28]. Moreover, via the downregulated expression of PTEN, the growth of gastric cancer can be promoted by the low expression of NBAT-1, as revealed by Yuan Gao [29]. In hepatocellular carcinoma, NBAT-1 suppresses the stability of c-Myc mRNA by binding to IGF2BP1 and inhibiting their interaction, thereby inhibiting tumourigenesis through cell cycle blockade and increased apoptosis [32]. In colorectal carcinoma (CRC), Chen Li proved the inhibition effect of lncRNA NBAT-1 on the development of OXA-resistant CRC cells by suppressing miR-4504 to mediate the WWC3/LATS1/YAP axis [34].

Several studies have revealed the link between NBAT-1 and urinary system. In bladder cancer, Dan Du revealed that NBAT-1 suppressed cell proliferation by the EMT pathway [33]. In renal carcinoma, NBAT-1 takes advantage of the miR-346/GSK-3β axis and inhibits cell migration and proliferation [36].

Additionally, for other systems, NBAT-1 can affect the prognosis of other cancers.—In epithelial ovarian cancer (EOC), Changsheng Yan verified that NBAT-1 acts as an anti-oncogene and can be potentially used in EOC therapy by targeting the ERK1/2 and AKT signalling pathways [31]. According to Chunhua Tian, in endometrial cancer, NBAT-1 can limit the invasion, migration, and proliferation of EC cells and facilitate apoptosis through PTEN regulation using the sponging miR-21-5p [35]. In breast cancer, Pengnan Hu verified that the cell invasion of breast cancer can be inhibited by NBAT-1 through the suppression of the EZH2-induced H3K27me3 of DDK1 mechanically [30]. As a key part of Polycomb Repressive Complex 2 (PRC2), EZH2 is found upregulated in several cancers and is related to epigenetic regulation [38]. In osteosarcoma, Cheng Yang demonstrated that NBAT-1 negatively modulates growth and metastasis, and invasion by physically interacting with miR-21 and then regulating downstream targets of miR-21, which includes RECK, TPM1, PDCD4, and PTEN [27].

It is proved that aberrant NBAT-1 expressions are likely to affect human cancer prognosis. But most existing research focuses on sample size and discrete outcomes. Thus, a quantitative meta-analysis was performed to elucidate the prognostic value of lncRNA NBAT-1 expression in cancer patients.

## Methods

### Literature search strategies

Using Web of Science and PubMed, two researchers independently identified relevant studies to explore the association between the pathological features of human cancers and NBAT-1 expression levels. The strategy to search literature combined multiple keywords (“cancer or carcinoma or tumour or neoplasm or malignant neoplasm or malignancy”, “NBAT1”, and “NBAT-1”). Together with these, identification of the obtained literature was performed to detect supplementary research works.

### Inclusion and exclusion criteria

The involved research should conform with the following inclusion criteria: 1) stated NBAT-1 expression levels according to the quantitative reverse transcription polymerase chain reaction (RT-qPCR); 2) offered the outcomes; 3) used specific standard of NBAT-1 expression levels to categorize patients into subgroups with low expression and high expression; 4) offered data on patients’ clinicopathological attributes, including at least one of the following ones: distant metastasis, histological grade, tumour invasion depth, lymph node metastasis, and TNM stage; 5) it should be a cohort or case–control research.

A study was not considered for analytical purposes if satisfy one of the following exclusion criteria: 1) stated recurring studies or included patients from an earlier research work; 2) did not provide sufficient data detail; 3) used non-human specimens; 4) were reports published in a non-English language; 5) were commentaries, reviews, unpublished data, and letters.

Concerning the exclusion and inclusion criteria, two researchers reviewed the title, abstract, and content of these studies to evaluate the research quality.

### Literature screening and data extraction

In the light of the inclusion and exclusion criteria, data were collected by two investigators (Yang Yu and Kedi Fan), and additionally, discrepancies were resolved via discussion or consensus with a third investigator (Yang Yu and Kedi Fan) prior to the analysis. Data derivation from previous research was: year of publication, first author, country of origin, types of cancer, identification method, the aggregate number of patients, number of patients in groups with low and high NBAT-1 expressions, and cut-off NBAT-1 approximate values at multiple expression levels.

### Quality assessment

The quality of related research was evaluated by the Newcastle‒Ottawa Scale, whose indexes include selection (4 points), results (3 points), and comparability (2 points), with scores ranging from 0 to 9. Two investigators carried out independent evaluation of the selected studies (Yang Yu and Kedi Fan) by resolving the differences via discussion or consensus with a third investigator (Yang Yu and Kedi Fan). Table 1 shows the score of each and every included research study. The higher the score, the better the methodological quality.

### Statistical analysis

Cochran’s and ChiI-square-based Q and I2 tests were employed to examine the heterogeneity of implicated studies. With the significance level of α = 0.1, the homogeneity test was conducted. *P* values < 0.5 were defined significant, and I2 values > 50% indicated that the studies are heterogeneous. a random effect model was employed for analysis purposes, and a fixed effect model to analyse homogeneous data. Statistical analysis was performed with StataSE 12.0 (Stata Corp LP, College Station, Texas, USA).

## Results

### Data selection and features

Twelve studies adhered to the inclusion criteria, involving 1600 patients. And all of the studies stemmed from China, wherein 3 studies were about non-small cell lung cancer [25, 26, 39], and the others about neuroblastoma [20], glioblastoma [24], osteosarcoma [27], gastric cancer [28], breast cancer [30], ovarian cancer [31], hepatocellular carcinoma [32], and bladder cancer [33], clear cell renal cell carcinoma [40].

**Fig. 1:**
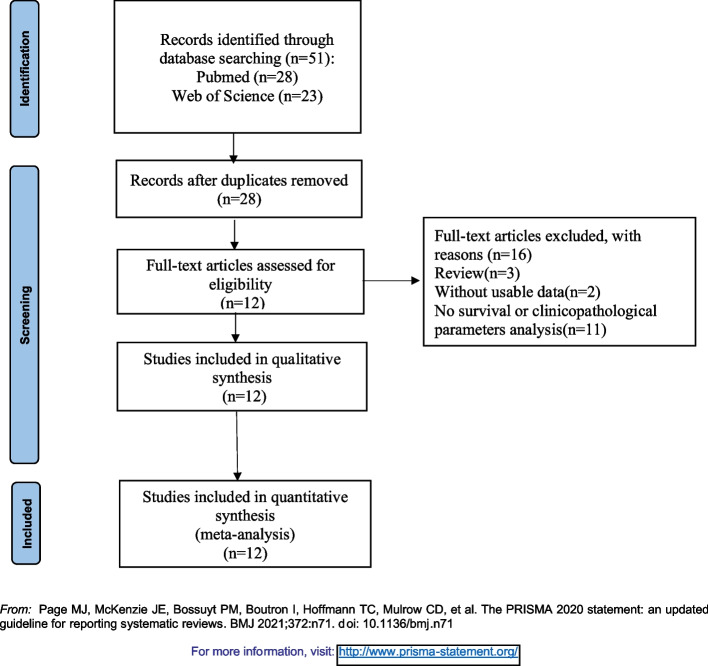
Flowchart of selecting studies for inclusion

RT qPCR was employed to record the NBAT-1 expression. On this basis, patients were classified into subgroups with low and high NBAT-1 expression. The mean, median, fold-change, and median ratio of relative NBAT-1 expression were used as cut-offs for estimating NBAT-1 expression. Table 2 presents the summaries of the attributes of related research, and the flow chart of the search and selection of research work is shown in Fig. 1.

**Table 2:** Characteristics of the included studies

Surname(Year)	Country	Cancer type	Total number	High	Low	Cut-off(high/low)	Detection method	Quality score
T Lei(2018 April) [25]	China	non-small cell lung cancer	69	34	35	mean	qRT‒PCR	6
TL Zheng(2018 September) [26]	China	non-small cell lung cancer	60	30	30	median	qRT‒PCR	8
DL Wang(2020 July) [39]	China	non-small cell lung cancer	162	80	82	mean	qRT‒PCR	6
J Liu(2017 May) [24]	China	glioblastoma	48	24	24	median	qRT‒PCR	7
C Yang(2017 October) [27]	China	osteosarcoma	60	30	30	median	qRT‒PCR	8
JJ Yan(2018 November) [28]	China	gastric cancer	78	34	44	fold-change = 2	qRT‒PCR	7
S Xue(2015 April) [40]	China	clear cell renal cell carcinoma	98	49	49	median	qRT‒PCR	6
PN Hu(2015 August) [30]	China	breast cancer	716	555	161	NA	qRT‒PCR	7
CS Yan(2017 April) [31]	China	ovarian cancer	57	28	29	median	qRT‒PCR	7
L Wei(2021 January) [32]	China	hepatocellular carcinoma	80	40	40	median	qRT‒PCR	7
Gaurav Kumar Pandey(2014 November) [20]	Sweden	neuroblastoma	93	50	43	NA	qRT‒PCR	9
Dan Du(2017 June) [33]	China	bladder cancer	79	45	34	median ratio of relative NBAT1 expression = 0.5	qRT‒PCR	6

### Correlation between NBAT-1 expression and pathological features

#### Age

**Fig. 2:**
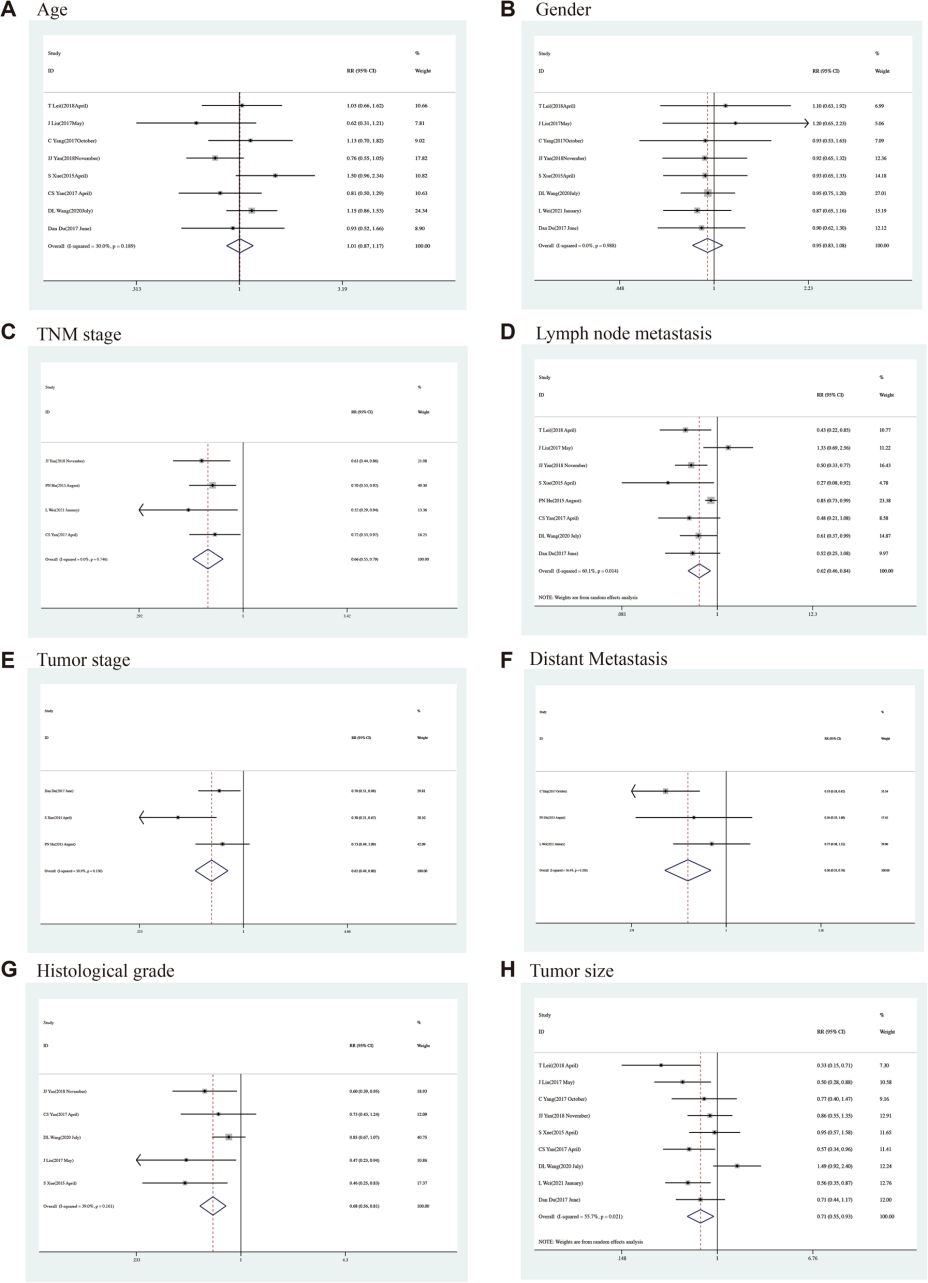
Forest plots for association of NBAT-1 expression with clinicopathological features. **A** Age. **B** Gender. **C** TNM stage. **D** Lymph node metastasis. **E** Tumor stage. **F** Distant Metastasis. **G** Histological grade. **H** Tumor size

Eight researches studied the correlation between Age (elderly vs. nonelderly) and the lncRNA NBAT-1 expression. In these studies, there was no statistically significant heterogeneity (*P* = 0.19, *I*^2^ = 30.00%). Therefore, it was not statistically significant to calculate the accumulated pooled RR and its 95% CI by using a fixed effect model [RR = 1.01, 95% CI (0.87,1.17), *P* = 0.93] (Fig. 2A, Table 3). The outcome suggested that the NBAT-1 expression level was independent of age.

**Table 3:** Meta-analysis results for the association of NBAT-1 with clinicopathological parameters

Clinicopathological parameters	Studies(n)	Numbers of patients	RR (95% CI)	*P* value	Heterogeneity		
					I2	Ph	Model
Age (elderly vs. nonelderly)	8	651	1.01(0.87,1.17)	0.93	30.00%	0.19	Fixed effects
Gender (male vs. female)	8	674	0.95(0.83,1.08)	0.40	0.00%	0.99	Fixed effects
TNM stage (III/IV vs. I/II)	4	912	0.66(0.55,0.79)	0.00	0.00%	0.75	Fixed effects
Lymph node metastasis (+ vs. -)	8	1303	0.62(0.46,0.84)	0.00	60.10%	0.01	Random effects
Tumour depth invasion(T3/4 vs. T1/T2)	3	890	0.62(0.49,0.80)	0.00	50.90%	0.13	Fixed effects
Distant Metastasis(+ vs. -)	3	806	0.50 (0.33,0.76)	0.00	36.40%	0.21	Fixed effects
Histological grade(poor/others vs. well/moderate)	5	443	0.68(0.57,0.81)	0.00	39.00%	0.16	Fixed effects
Tumour size	9	731	0.72(0.55,0.93)	0.01	55.70%	0.02	Random effects

#### Gender

The relationship between gender (male vs. female) and NBAT-1 expression was discussed in eight studies, which showed heterogeneity with no statistical significance (*P* = 0.99, *I*^2^ = 0.00%). Consequently, a fixed-effect model was employed to calculate the pooled RR with its 95% CI, resulting in a non-statistically significant outcome [RR = 0.95, 95% CI (0.83,1.08), *P* = 0.40] (Fig. 2B, Table 3). As revealed by it, the NBAT-1 expression level was independent of gender.

#### TNM stage

Four studies indicated the link between the TNM stage (III/IV vs. I/II) and lncRNA NBAT-1 expression. No statistically significant heterogeneity (*P* = 0.75, *I*2 = 0.00%) was obtained. Accordingly, the pooled RR was calculated with its 95% CI using a fixed effect model, and significant differences were obtained [RR = 0.66, 95% CI (0.55, 0.79), and *P* = 0.00] (Fig. 2C, Table 3). It is suggested that the advanced TNM stage may appear at a low expression level.

#### Lymph node metastasis

There was altogether eight research focused on the relationship between lymph node metastasis and NBAT-1 expression, with statistically significant heterogeneity (*P* = 0.01, *I*2 = 60.10%). Therefore, the pooled RR with a 95% CI was calculated by a random effect model, and a significant difference [RR = 0.62, 95% CI (0.46, 0.84), and *P* = 0.00] (Fig. 2D, Table 3) was achieved. As illustrated, the group with low NBAT-1 expression level tended to have a higher risk of lymph node metastasis than the high NBAT-1 expression level group.

#### Tumour invasion depth (T)

Three studies explored the relationship between the depth of tumour invasion and the expression of NBAT-1 with no statistical significance (*P* = 0.13, *I*2 = 50.90%). Accordingly, the application of a random-effect model was made to calculate the pooled RR, with a 95% CI. A significant difference was recorded [RR = 0.62, 95% CI (0.49,0.80), and *P* = 0.00] (Fig. 2E, Table 3). Compared with the highly expressed NBAT-1 group, the group with NBAT-1 low expression level appeared riskier in deep tumour attack (T2 stage or above).

#### Distant metastasis

Three studies showed the link between distant metastasis and NBAT-1 expression levels, which indicated heterogeneity with no statistical significance (*P* = 0.21, *I*2 = 36.40%). Accordingly, the pooled RR, with its 95% CI, was calculated by using a fixed effect model, and significant differences were obtained [RR = 0.50, 95% CI (0.33, 0.76), and *P* = 0.00] (Fig. 2F, Table 3). The group with low NBAT-1 expression levels possessed a higher risk of vascular invasion than the group with high NBAT-1 expression levels.

#### Histological grade

Five studies explored the relationship between histological grade and NBAT-1 expression. In the studies considered, no statistically significant heterogeneity was recorded (*P* = 0.16, I2 = 39.00%). Therefore, the pooled RR, with its 95% CI, was calculated by using a fixed effect model, and significant differences were obtained [RR = 0.68, 95% CI (0.57, 0.81), and *P* = 0.00] (Fig. 2G, Table 3). It is indicated that the low expression of NBAT-1 is significantly correlated with the advanced histological grade (poor vs. well/moderate).

#### Tumour size

A total of nine studies have found a link between tumour size and NBAT-1 expression, with no statistically significant heterogeneity (*P* = 0.02, I2 = 55.70%). Consequently, the pooled RR, with its 95% CI, was calculated by using a random effect model, and a significant difference was obtained [RR = 0.72, 95% CI (0.55, 0.93), and *P* = 0.01] (Fig. 2H, Table 3). It is indicated that compared with the group with high NKILA expression level, the group with low NKILA expression level was easily affected by the increased risk of tumour size.

### Association between NBAT-1 expression and survival in various cancer types

**Fig. 3:**
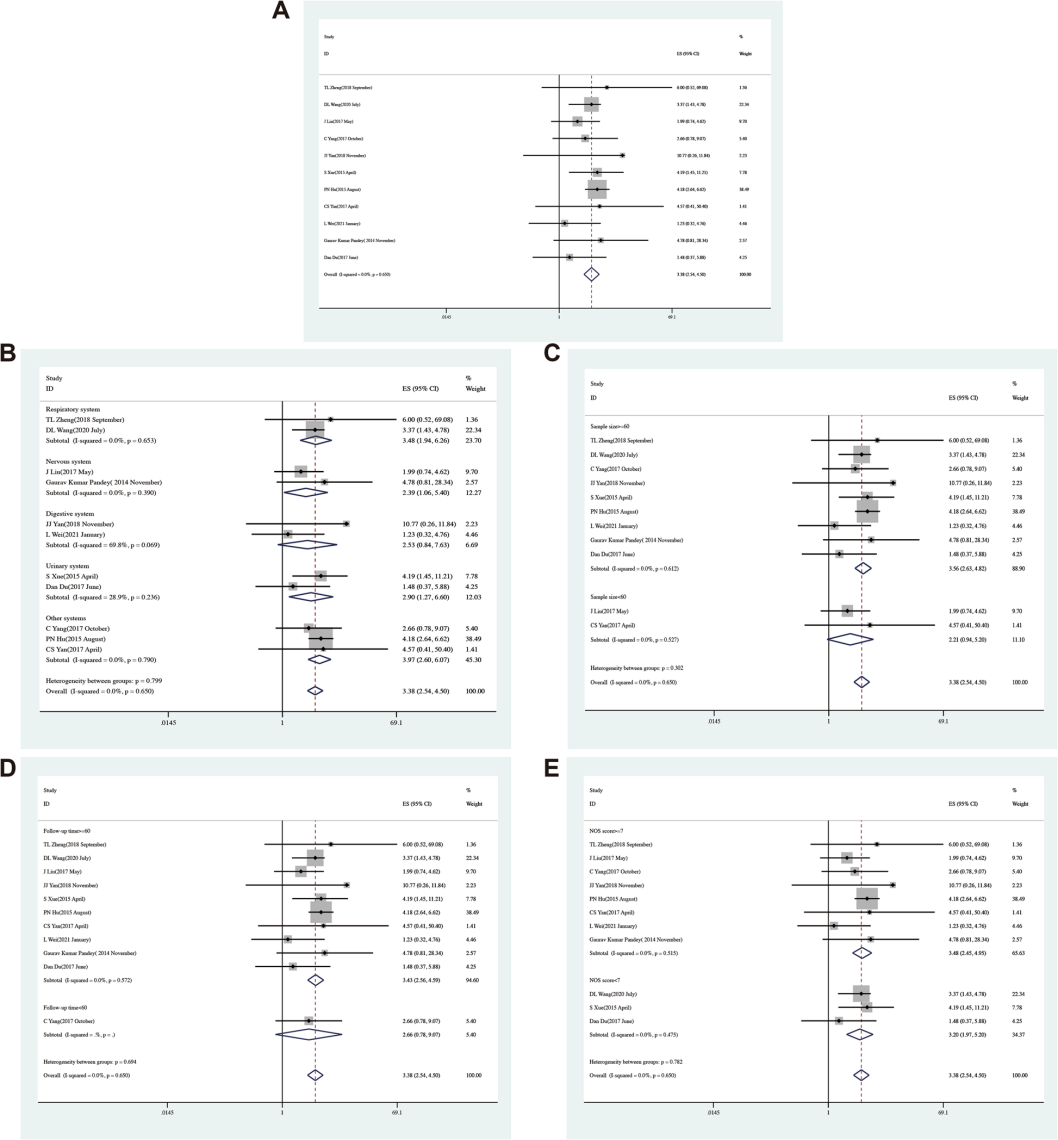
Relationship between NBAT-1 expression and overall survival. **A** Forest plots for association of NBAT-1 expression with overall survival. **B** Subgroup analysis stratified by cancer type. **C** Subgroup analysis stratified by sample size. **D** Subgroup analysis stratified by follow-up time. **E** Subgroup analysis stratified by NOS score

Eleven studies, which involved 1531 patients were analysed to assess the impact of NBAT-1 expression on OS in multiple kinds of cancers (Table 4). It is revealed that the decreased expression of NBAT-1 indicated that the OS performance of related cancers was weak [pooled HR = 3.38, 95% CI (2.54, 4.50), *P* = 0.00], with heterogeneity (I2 = 0.00%, *P* = 0.65) (Fig. 3A). According to different tumours in the nervous system, urinary system, respiratory system, and digestive system (Fig. 3B), the researchers measured the sample size (*n* ≥ 60 or *n* < 60) (Fig. 3C), follow-up time (≥ 60 months or < 60 months) (Fig. 3D), and NOS score (NOS scores ≥ 7 or < 7) (Fig. 3E). Compared with the group with high NBAT-1 expression, a statistically significant OS reduction and poor survival rate were discovered in the group with low NBAT-1 expression, as depicted in Table 5.

**Table 4:** Characteristics of the overall survival of patients in the included studies

Surname(Year)	Country	Cancer type	Survival analysis	HR statistic	Hazard ratios	Lower 95% CI	Higher 95% CI	Follow-up months	Outcome
TL Zheng(2018 September)	China	non-small cell lung cancer	Univariate	survival curves	6.00	0.52	69.08	60	OS
DL Wang(2020 July)	China	non-small cell lung cancer	Univariate/Multivariate analysis	data in paper	3.37	1.43	4.78	60	OS
J Liu(2017 May)	China	glioblastoma	Univariate	survival curves	1.99	0.74	4.62	60	OS
C Yang(2017 October)	China	osteosarcoma	Univariate	survival curves	2.66	0.78	9.07	50	OS
JJ Yan(2018 November)	China	gastric cancer	Univariate	survival curves	10.77	0.26	11.84	100	OS
S Xue(2015 April)	China	clear cell renal cell carcinoma	Univariate/Multivariate analysis	data in paper	4.19	1.45	11.21	60	OS
PN Hu(2015 August)	China	breast cancer	Univariate	survival curves	4.18	2.64	6.62	60	OS
CS Yan(2017 April)	China	ovarian cancer	Univariate	survival curves	4.57	0.41	50.40	60	OS
L Wei(2021 January)	China	hepatocellular carcinoma	Univariate	survival curves	1.23	0.32	4.76	60	OS
Gaurav Kumar Pandey(2014 November)	Sweden	neuroblastoma	Univariate	survival curves	4.78	0.81	28.34	300	OS
Dan Du(2017 June)	China	bladder cancer	Univariate	survival curves	1.48	0.37	5.88	60	OS

**Table 5:** Subgroup meta-analysis of pooled HRs for OS

Stratified analysis	Studies (n)	Number of patients	Pooled HR (95% CI)	*P*-value	Heterogeneity		
					I^2^%	*P*-value	Model
NOS score							
≥ 7	8	1192	3.480 (2.447,4.948)	< 0.001	0.0%	0.515	Fixed
< 7	3	339	3.198 (1.966,5.201)	< 0.001	0.0%	0.475	Fixed
Follow-up time							
≥ 60	10	1471	3.427(2.556,4.595)	< 0.001	0.0%	0.572	Fixed
< 60	1	60	2.660(0.780,9.070)	0.118			
Cancer type							
Respiratory system	2	222	3.483 (1.939,6.258)	< 0.001	0.0%	0.653	Fixed
Nervous system	2	141	2.392 (1.060,5.398)	0.036	0.0%	0.390	Fixed
Digestive system	2	158	10.770 (0.260,11.840)	0.098	69.8%	0.069	Fixed
Urinary system	2	177	2.900 (1.275,6.600)	0.011	28.9%	0.236	Fixed
Other systems	3	833	3.972 (2.600,6.067)	< 0.001	0.0%	0.790	Fixed
Sample size							
≥ 60	9	1426	3.564 (2.634,4.823)	< 0.001	0.0%	0.612	Fixed
< 60	2	105	3.380 (2.542,4.496)	0.069	0.0%	0.527	Fixed

### Assessment of publication bias

**Fig. 4:**
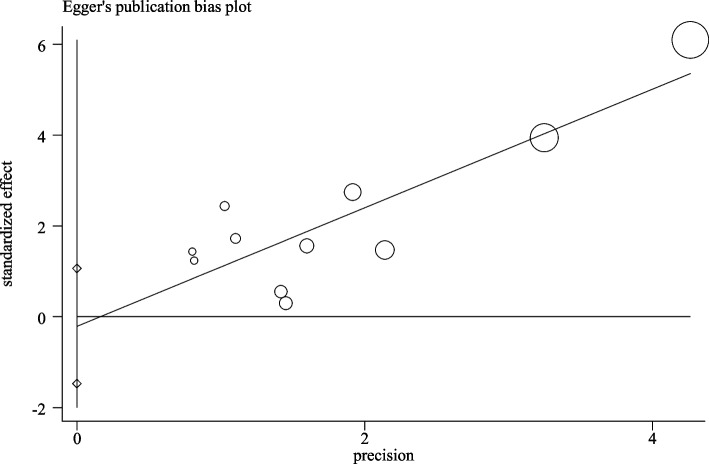
Egger’s test for publication bias of results of overall survival (OS)

As for the publication bias, Egger’s funnel plot was employed in this research. There was no considerable publication bias in Age (*P* = 0.62), Gender (*P* = 0.13), TNM stage (*P* = 0.12), distant metastasis (*P* = 0.67), lymph node metastasis (*P* = 0.07), tumour size (*P* = 0.18), tumour stage (*P* = 0.34) and original OS (*P* = 0.72) (Fig. 4). However, considerable publication bias was observed in histological grading analysis (*P* = 0.03).

### Assessment of sensitivity

**Fig. 5:**
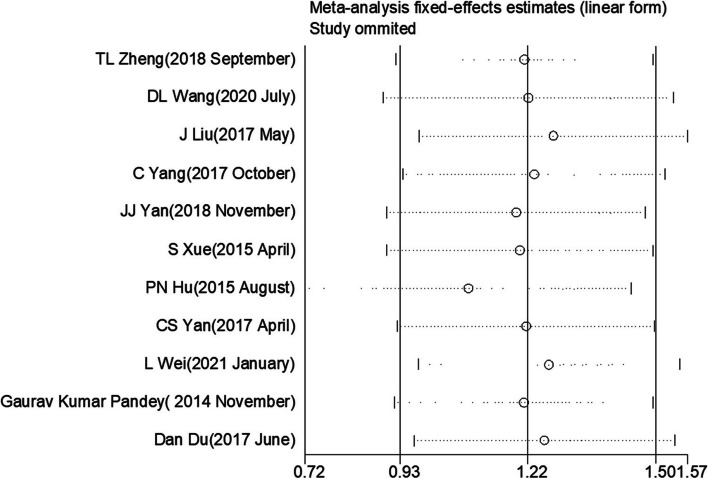
Sensitivity analysis for studies on OS by omitting each study sequentially

To evaluate the stability of the initial OS data, the researcher conducted a sensitivity analysis. The meta-analysis results were robust since when any study was excluded, they remain stable (Fig. 5).

## Discussion

Recently, research indicated that long noncoding RNAs (lncRNAs) are fundamental in diagnosing and treating cancers [41, 42]. Additionally, dysregulation of lncRNAs is related to the development of cancer, due to their ability to regulate alternative splicing and translation, stabilise the host mRNAs in post-transcriptional phenomena, or act as a scaffold or guide to regulate protein-DNA or protein–protein interactions [4–6, 43]. These lncRNAs with atypical expression are considered molecular biomarkers that will promote the growth of diagnosis and prognosis strategies for different types of human cancers [44].

This meta-analysis in human cancers was about how the expression level of NBAT-1 influences pathological attributes. There were 1600 patients from 12 studies involved. The fixed effect model was suitable for the analysis of age, gender, TNM stage, tumour invasion depth, distant metastasis and histological grade. Furthermore, random effects were applied for tumour size, and lymph node metastasis. Therefore, the low NBAT-1 expression level group possessed a higher risk of deep tumour invasion, distant metastasis, lymph node metastasis, poorly distinguished histological grade, advanced TNM stage, and large tumour size than the low NBAT-1 expression level group. Furthermore, the NBAT-1 expression level is independent of gender and age.

Nevertheless, this research work contained some limitations: (1) each and every research work included in this study originated from China, excluding patients from other states; (2) due to the relatively small scale of patients registered, the investigated cancer types were incomprehensive; (3) consensus failed to be reached on the cut-off approximated in order to distinguish between the groups with low or high NBAT-1 expression level; (4) there was no cohort research work satisfying the inclusion criteria. Therefore, to validate the results, high-quality research with a considerable sample size is required.

Briefly, the low expression level of lncRNA NBAT-1 had a close association with distant metastasis, deep tumour invasion, lymph node metastasis, poorly distinguished histological grade, advanced TNM stage, and large tumour size, suggesting that it may serve as a biomarker for cancer patients with poor prognosis.

## Conclusion

As discovered in this meta-analysis, reduced expression of NBAT-1 can predict poor prognosis in several cancers, demonstrating that NBAT-1 can serve as a promising prognostic factor of human cancers. AND the low expression level of lncRNA NBAT-1 had a close association with distant metastasis, deep tumour invasion, lymph node metastasis, poorly distinguished histological grade, advanced TNM stage, and large tumour size, suggesting that it may serve as a biomarker for cancer patients with poor prognosis Nevertheless, so far, it remains crucial to conduct better-designed studies with larger scales to confirm our findings.

## Data Availability

All data generated or analysed during this study are included in this published article.

## Abbreviations

ncRNAs: Noncoding RNAs
lncRNAs: Long noncoding RNAs
CRC: Colorectal carcinoma
EOC: Epithelial ovarian cancer
PRC2: Polycomb Repressive Complex 2
RT-qPCR: Quantitative reverse transcription polymerase chain reaction
NOS: The Newcastle–Ottawa Scale
OS: Overall survival
